# Isolation and Characterization of Neural Crest-Derived Stem Cells From Adult Ovine Palatal Tissue

**DOI:** 10.3389/fcell.2018.00039

**Published:** 2018-04-11

**Authors:** Marie-Theres Zeuner, Nikolai N. Didenko, David Humphries, Sokratis Stergiadis, Taryn M. Morash, Ketan Patel, Wolf-Dieter Grimm, Darius Widera

**Affiliations:** ^1^Stem Cell Biology and Regenerative Medicine, School of Pharmacy, University of Reading, Reading, United Kingdom; ^2^Stem Cell Lab, Department for Personalized Medicine, Scientific Innovation Centre, Stavropol State Medical University, Stavropol, Russia; ^3^Centre for Dairy Research, School of Agriculture, Policy and Development, University of Reading, Reading, United Kingdom; ^4^Animal, Dairy and Food Chain Sciences Research Group, Centre for Dairy Research, School of Agriculture, Policy and Development, University of Reading, Reading, United Kingdom; ^5^Skeletal Muscle Development Group, School of Biological Sciences, University of Reading, Reading, United Kingdom; ^6^Periodontology, Department of Dental Medicine, Faculty of Health, University of Witten/Herdecke, Witten, Germany

**Keywords:** neural crest-derived stem cells, neural crest, multipotency, ovine stem cells, large animal model

## Abstract

Adult mammalian craniofacial tissues contain limited numbers of post-migratory neural crest-derived stem cells. Similar to their embryonic counterparts, these adult multipotent stem cells can undergo multi-lineage differentiation and are capable of contributing to regeneration of mesodermal and ectodermal cells and tissues *in vivo*. In the present study, we describe for the first time the presence of Nestin-positive neural crest-derived stem cells (NCSCs) within the ovine hard palate. We show that these cells can be isolated from the palatal tissue and are able to form neurospheres. Ovine NCSCs express the typical neural crest markers Slug and Twist, exhibit high proliferative and migratory activity and are able to differentiate into α smooth muscle cells and β-III-tubulin expressing ectodermal cells. Finally, we demonstrate that oNCSCs are capable of differentiating into osteogenic, adipogenic and chondrogenic cells. Taken together, our results suggest that oNCSCs could be used as model cells to assess the efficacy and safety of autologous NCSC transplantation in a large animal model.

## Introduction

Emerging evidence suggests that adult craniofacial tissues in vertebrates contain limited numbers of post-migratory NCSCs (reviewed in Kaltschmidt et al., 2012). Adult multipotent NCSCs possess high levels of cellular plasticity which is only surpassed by pluripotent stem cells including embryonic stem cells and induced pluripotent stem cells. In particular, NCSCs have been shown to differentiate efficiently into ectodermal and mesodermal progeny including neuronal, glial, osteogenic, adipogenic, and chondrogenic cells, as well as melanocytes and mesenchymal stem cells (MSCs).

Previously, we have shown that multipotent Nestin-expressing NCSCs are present within the rat, mouse, and human palate (Widera et al., 2009; Martin et al., 2012). Anatomically, within the sub-palatal lamina propria, these cells are localized adjacent to specialized mechanoreceptors referred to as Meissner corpuscles (reviewed in Widera et al., 2012).

Being a highly regenerative tissue (Kahnberg and Thilander, 1982, 1984, 1987), the mammalian palatal tissue contains several other cell types harboring progenitor cell and stem cell properties in addition to NCSCs. In particular, the palate is known to contain mesenchymal stem cells and neural crest-derived Schwann cell progenitor cells (reviewed in (Widera et al., 2012). Especially the latter cell type is known to be able to revert its cellular phenotype, to re-enter the cell cycle and to differentiate into ectodermal and mesodermal cells of the cranial neural crest progeny.

Palatal NCSCs can be accessed within minimally-invasive surgical procedures, expanded *in vitro* as self-adherent neurospheres under serum-free conditions and are able to differentiate into major ectodermal and mesenchymal neural crest derivatives. Thus, NCSCs represent promising candidate cells for the use within modern regenerative medicine (Grimm et al., 2014). However, future use of human NCSCs in the clinical routine requires rigid testing in appropriate animal models to evaluate their safety and efficacy. Notably, according to the revised ISSCR guidelines for stem cell research and clinical translation, large animal models should be used since they better emulate the human anatomy and/or pathology (Daley et al., 2016). In particular, the route of administration, the optimal cell numbers and/or dose of biologicals can be better extrapolated to the human system based on large animal studies. In this context, sheep models offer the advantage of a body weight similar to humans and a significantly longer lifespan than rodent models allowing long term studies. This is especially important in evaluating the safety and efficacy of autologous stem cell transplantation in long term preclinical studies.

Several adult stem cell populations have been identified in the sheep including amniotic fluid MSCs (Tian et al., 2016), MSCs derived from peripheral blood (Lyahyai et al., 2012), MSCs from the olfactory epithelium (Veron et al., 2018) and the neural crest-derived periodontal ligament stem cells (Gronthos et al., 2006).

In the present study, we hypothesized that the ovine palate contains NCSCs imbued with a developmental potential equivalent to their rodent and human counterparts. We characterized the endogenous niche of ovine NCSCs (oNCSCs) histologically and immunohistochemically and successfully isolated and cultivated the cells as adherent monolayer and as self-adherent neurosphere cultures. Cultivated oNCSCs were highly proliferative with an average population doubling time of 26 h. We showed that oNCSCs express typical neural crest markers and are migratory. Finally, we demonstrated that oNCSCs differentiate into ectodermal and mesodermal neural crest derivatives.

## Materials and methods

### Ovine neural crest-derived stem cell (oNCSCs) isolation and culture

Palatal tissue was extracted from female sheep according to local guidelines and with approval from the Stavropol State Medical University Animal Ethics Committee (approval number 39, 16/04/2014). Prior to surgery, animals were starved overnight and had antibiotics administered (penicillin/streptomycin 3 ml/kg i.m) and general anesthesia was induced with thiopentone 20 mg/kg i.v. The sheep were intubated orally and anesthesia was maintained by halothane (1–2%) and nitrous oxide/oxygen in a ratio of 1:2. Extraction of the tissue was achieved using minimally-invasive access to the palate followed an atraumatic approach. For harvesting the stem cell-containing palatal tissue, a horizontal incision to the bone has been made 5 mm from the palatal gingival margin. Another horizontal incision has been made 2 mm coronal to the first incision and the periosteum has been dissected before removing the wedge of soft tissue. An approximately 10 × 6 mm subepithelial connective tissue graft was harvested from the palate and placed in 100 ml pre-cooled ChillProtec® plus solution (Merck Millipore, Germany, Charge 0088D). Incisions were closed with resorbable sutures (VICRYL Plus 3-0, 70 36, Ethicon, Germany). An antiseptic mouthwash was applied after surgery (chlorhexidine, 0.2%) for 3 days, and animals were returned to normal grazing. Additional control palatal tissue was extracted post-mortem at the Centre for Dairy Research (The University of Reading, Hall Farm) according to local guidelines.

After mechanical dissection using a scalpel and surgical scissors, the palatal tissue was dissociated using 4 mg/ml Dispase (Sigma-Aldrich) at 4°C for overnight followed by Collagenase I (Sigma-Aldrich; 0.3 units/ml, 90 min, 37°C) treatment and mechanical trituration with a fire-polish glass Pasteur pipette. oNCSCs were cultured in serum-free media (Dulbeccos modified Eagles medium [DMEM]/F12; Sigma-Aldrich) containing basic FGF-2 (20 ng/ml; Miltenyi Biotec, Bergisch Gladbach, Germany), EGF (20 ng/ml; Peprotech, London, United Kingdom), B27 supplement (Thermo-Fisher, Paisley, United Kingdom) in low adhesion T25 cell culture flasks (Sarstedt) in a humidified incubator at 37°C and 10% CO_2_. After 8–10 days, the cultures reached sub-confluency and primary neurospheres were dissociated using Trypsin/EDTA (Sigma-Aldrich) and a 100 μm cell strainer (BD Falcon, Swindon, United Kingdom) resulting in an average of 2 × 10^6^ single oNCSC per flask. The resulting oNCSC suspension was resuspended in DMEM/F12 (Sigma-Aldrich) containing basic FGF-2 (20 ng/ml; Miltenyi Biotec), EGF (20 ng/ml; Peprotech), B27 supplement (Thermo-Fisher, Paisley, United Kingdom) and 10% newborn calf serum (Sigma-Aldrich). The sub-culturing protocol consisted of neurosphere passaging every 3–4 days with whole culture media change. For adherent culture, neurospheres were dissociated and cultivated in DMEM/F12 (Sigma-Aldrich) supplemented with 10% fetal calf serum (FCS, Sigma-Aldrich), FGF-2 (20 ng/ml; Miltenyi Biotec), and EGF (20 ng/ml; Peprotech). Medium was changed every 2–4 days. Adherent cells were passaged at 80–90% confluency using Trypsin/EDTA.

### Preparation of cryosections

The mucoperiosteum was embedded in O.C.T compound (TAAB Laboratory Equipment Ltd., Reading, UK) and quick-frozen in super-cooled ethanol (−80°C). Cryostat sections (10 μm, Bright OT5000 Cryostat) were cut and mounted on poly-lysine-coated glass slides (VWR International Ltd., Lutterworth, UK) and processed for immunocytochemistry as stated below.

### Hematoxylin and eosin stain

Palatal cryostat sections were prepared as described above and dried at room temperature for 15 min. Subsequently, sections were briefly washed in PBS and stained in Mayers Haematoxylin solution (Sigma-Aldrich). After the sections were washed with distilled H_2_O (d H_2_O), slices were incubated in 70% acidic alcohol (70% ethanol (Sigma-Aldrich) with 0.1% HCl (Fisher scientific) for 30 s, before slices were washed in dH_2_O for 5 min. Counterstaining with 1% Eosin Y (Eosin Y, 5% wt/vol solution in water, Sigma-Aldrich) in 70% ethanol was carried out for 2 min. Stained slices were dehydrated using incubation in increasing ethanol concentrations (70%, 1 min; 90%, 2 min; 100% 3 times 2 min. Xylene was applied for 2 × 3 min, and sections were mounted in Mowiol (Sigma-Aldrich). The sections were examined using Zeiss AxioSkop system (Carl Zeiss, Jena, Germany) with Axiovision4 software (Carl Zeiss).

### Immunocytochemistry

oNCSCs were cultured on tissue culture-treated glass coverslips (VWR) and fixed using phosphate-buffered 4% paraformaldehyde (PFA, pH 7.4) (4% wt/vol PFA, 100 mM NaH_2_PO_4_, Sigma-Aldrich) for 20 min at 4°C followed by three washing steps in 1 × PBS for 5 min. Blocking was done in 5% goat serum (Stratech Scientific Unit, Suffolk, UK) for 30 min at 23°C followed by incubation with primary antibodies anti-Nestin, 1:500 (RnD Systems, Abingdon, UK); anti-β-III-tubulin, 1:200 (Promega, Madison, WI,USA); anti-smooth muscle actin (αSMA), 1:100 (Chemicon, EMD Millipore, Merck Group, Darmstadt, Germany) for 1 h at 23°C. The secondary fluorochrome-conjugated antibodies were diluted 1:300 (Alexa 488, 1 h at 23°C; Life Technologies Ltd., Paisley, UK). Nuclear counterstaining was performed with DAPI (1:2000 in PBS; Sigma Aldrich). Antibody staining was visualized using epifluorescence microscope (Zeiss AxioImager system, Carl Zeiss) and Axiovision4 software (Carl Zeiss).

### Polymerase chain reaction (non-quantitative and quantitative)

Total RNA from oNCSCs was isolated using a NucleoSpin 8 core kit (Macherey-Nagel) according to manufacturer's guidelines. cDNA synthesis was performed using First Strand cDNA Synthesis Kit (Thermo Fisher Scientific), followed by PCR using DreamTaq Green PCR MasterMix (2x) (Thermo Fisher Scientific). The cycling conditions comprised an initial denaturation of 30 s at 95°C and 36 cycles of 10 s at 95°C, 20 s at the appropriate temperature, and 20 s at 72°C followed by final elongation for 5 min at 72°C. qPCR was performed using SYBR® Green PCR Master Mix (Thermo Fisher Scientific) and measured in a StepOnePlus qPCR Cycler (Applied Biosystems, Thermo Fisher Scientific) according to respective manufacturer's guidelines. Expression of stem cell-related genes was calculated relative to that of Actin. Primer sequences are provided in Table 1.

**Table 1:** Primer sequences for PCR.

Ovine *Actin* fwd	GGTTGGTGATGAGGCAAGTG
Ovine *Actin* rev	TGGTTGGGTTCATAGGAGGT
Ovine *Nestin* fwd	GGTCTGTGGAAGGGAACCAC
Ovine *Nestin* rev	TCCAAGCGCCTTAGACTTCC
Ovine *Slug* fwd	ATCTCCCCGTGTCTCTACGA
Ovine *Slug* rev	ATCCGGACAGAGGGGATAGG
Ovine *Twist* fwd	GAGCTGGACTCCAAGATGGC
Ovine *Twist* rev	CCATCCTCCAGACCGAGAAG

### Ectodermal differentiation

Ectodermal differentiation was induced as described in Müller et al. (2015) with minor modifications. Briefly, cells were pre-cultivated in DMEM/F12 (Sigma-Aldrich) supplemented with 20% FCS (Sigma-Aldrich) until full confluency was reached followed by a change of the medium to neuronal differentiation medium (DMEM high glucose, 200 mM l-glutamine, 10% FCS, 500 μM 3-isobutyl-1-methylxanthine, 200 μM indomethacin, 1 μM dexamethasone, and 2 μM insulin, all Sigma-Aldrich) and cultivated for subsequent 7 days. In the following, the neuronal differentiation medium was supplemented with 5 μM retinoic acid and the cells were cultivated for additional 7 days. For analysis, cell were fixed with phosphate-buffered 4% PFA (pH 7.4, Sigma-Aldrich) for 1 h at 4°C, and processed for immunocytochemistry as described above.

### Mesodermal differentiation

To induce mesodermal differentiation, secondary neurospheres were dissociated and resuspended in DMEM (Sigma-Aldrich) containing 10% FCS and plated at 1 × 10^5^ cells per well in 24-well cell culture plates. Medium was changed every 2–3 days. After 14 days of differentiation, cells were fixed and processed for immunocytochemical analysis as described above.

### Osteogenic differentiation

To induce osteogenic differentiation, 4 × 10^3^ cells/cm^2^ were seeded in normal cultivation medium. Medium was changed after 3 days to Stem Pro Osteocyte/Chondrocyte basal medium (Life Technologies) supplemented with StemPro Osteogenesis supplement (Life Technologies) according to the suppliers instructions. Cultures were maintained at 37°C and 5% CO_2_ for 7 days for alkaline phosphatase stain, or for 21 days for alizarin red staining. For alkaline phosphatase stain, alkaline phosphatase detection kit (Millipore, Merck group) was used according to manufacturer's guidelines. Briefly, media was removed and cells were fixed using 4% PFA for 2 min. Fixative was removed and cells were washed using TBST (20 mM Tris-HCl, pH 7.4, 0.15 M NaCl, 0.05% Tween-20). Fast red violet (FRV) was mixed with Napthol AS-B1 phosphate solution and water ratio 2:1:1). Staining solution was added to each well and incubated in the dark at room temperature for 15 min. Staining solution was removed and cells were rinsed using TBST. Cells were covered in PBS before image acquisition using Zeiss AxioSkop system (Carl Zeiss) with Axiovision4 software (Carl Zeiss). For alizarin red stain, medium was removed and cells were washed with PBS. Fixation in 4% PFA for 30 min was followed by three washing steps in dH_2_O. Alizarin red staining solution [2% wt/vol Alizarin Red S (Sigma-Aldrich) in dH_2_O, pH 4.2 with 0.1% NH4OH, filtered through a 0.2 μm filter (Sarstedt)] was added and incubated for 45 min at room temperature in the dark. The staining solution was removed by three washing steps with water. PBS was added before imaging using Zeiss AxioSkop system (Carl Zeiss) with Axiovision4 software (Carl Zeiss). To quantify the amount of deposited calcium, the different test substrates were immersed in 400 μl 10% acetic acid and incubated under shaking for 30 min at RT. After the monolayer was detached, the solution was heated to 85°C for 20 min. Subsequently, the samples were centrifuged at 20,000 × *g* for 15 min and the supernatant was transferred to a new test tube. Prior to photometry, the pH value was set to 4.3 and the optical density was measured at a wavelength of 405 nm using Spectra Max 340PC plate reader (Molecular Devices, Wokingham, UK). Dye recovery was determined using a standard curve.

### Adipogenic differentiation

1 × 10^4^ cells/cm^2^ were seeded in normal cultivation medium for 3 days before medium was changed to StemPro Adipogenic basal medium (Life Technologies) supplemented with StemPro Adipogenesis supplement (Life Technologies). Medium was replaced every 2–3 days. After 21 days at 37°C and 5% CO_2_, cells were processed for Oil red O stain. Plates were imaged using Zeiss A1 inverted epifluorescence microscope system (Carl Zeiss).

### Chondrogenic differentiation

1 × 10^6^ cells were cultured as pellet culture in StemPro Osteocyte/Chondrocyte basal medium (Life Technologies) supplemented with StemPro Chondrogenesis supplement (Life Technologies) in a humidified incubator at 37°C and 5% CO_2_. Fresh medium was provided every 2–3 days. After 21 days, medium was aspirated and cell pellets were washed with PBS before fixation in 4% PFA for 60 min. After 3 washings steps with ddH_2_O, alcian blue staining solution was added [0.1 mg/ml Alcian Blue dissolved in 6 parts EtOH and 4 parts of acetic acid (Sigma-Aldrich)] and incubated over night at room temperature under exclusion of light. Cell pellets were embedded in O.C.T compound (TAAB Laboratory Equipment Ltd., Reading, UK) and frozen in super-cooled ethanol at −80°C. Cryostat sections (10 μm) were prepared (Bright OT5000 cryostat), mounted on poly-lysine-coated glass slides (VWR) and visualized using a Nikon TiE microscope (Nikon UK Ltd., Kingston upon Thames, UK).

### Wound healing assay

oNCSCs were cultivated as adherent monolayer cultures until full confluency and a vertical scratch was made in each well using a small plastic pipette tip followed by a careful washing step with PBS. The medium was replaced with normal culture medium. Images were taken every 10 min with a Nikon TiE Time lapse System (Nikon UK Ltd.,). Image analysis was performed using ImageJ's Fiji (Schindelin et al., 2012).

### Proliferation assay

Neurospheres were dissociated as described above and 1.0 × 10^4^ oNCSCs/ml were cultivated in low adhesion cell cultures plates for a period of 4 days. Cell numbers were determined every 24 h by counting the total cell number using an improved Neubauer haematocytometer (VWR) after centrifugation at 212 × *g* for 5 min and dissociation of the neurospheres.

### Statistical analysis

Statistical analysis was performed using GraphPad Prism software (GraphPad, La Jolla, CA, USA). Student's *t*-test (two-tailed, confidence interval (CI) 95%), or one-way analysis of variance (ANOVA) followed by Bonferroni correction (CI 95%), was used where appropriate and *p* < 0.05 was considered statistically significant. At least 3 independent measurements were performed.

## Results

### Ovine hard palate contains nestin-positive NCSCs

**Figure 1:**
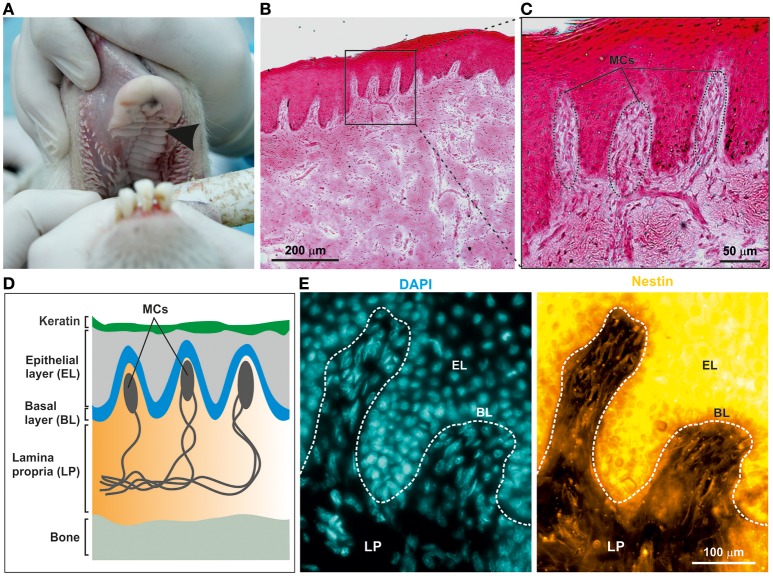
Adult ovine hard palate contains neural crest-derived stem cells. **(A)** Tissue was isolated from ovine hard palate and sagittal sections were made along palatal ridges. Arrow indicates a palatal ridge. **(B,C)** Sagittal sections along ovine palatal ridges stained with Hematoxylin and Eosin revealed structure of Meissner corpuscles (MCs) localized within the palatal ridges. Scale bars: **(B)** 200 μm; **(C)** 50 μm. **(D)** Schematic drawing of MCs within the submucosa of palatal ridges. MCs are localized in the lamina propria (LP) of the palatal ridges underneath the keratin layer, the epithelial layer (EL), and the basal layer (BL). **(E)** Immunohistochemical stainings revealed the presence of Nestin-expressing oNCSCs (yellow) in the LP, especially within MC. Nuclear counterstaining was performed using DAPI (cyan). Scale bar: 100 μm.

To examine the potential presence of NCSCs within the ovine palate and, more specifically, palatal ridges (Figure 1A, arrow), cryostat section of the mucoperiosteum were prepared. Hematoxylin and Eosin staining revealed the presence of numerous Meissner corpuscles within the lamina propria underneath the epithelial layer (Figures 1B–D). Notably, the Meissner corpuscles showed the typical high level of innervation (Figure 1C). An immunohistochemical staining against the neural crest marker Nestin revealed the presence of Nestin filaments within the Meissner corpuscles in the palatal ridges (Figure 1E).

### Isolated oNCSCs form neurospheres and express neural crest markers *in vitro*

**Figure 2:**
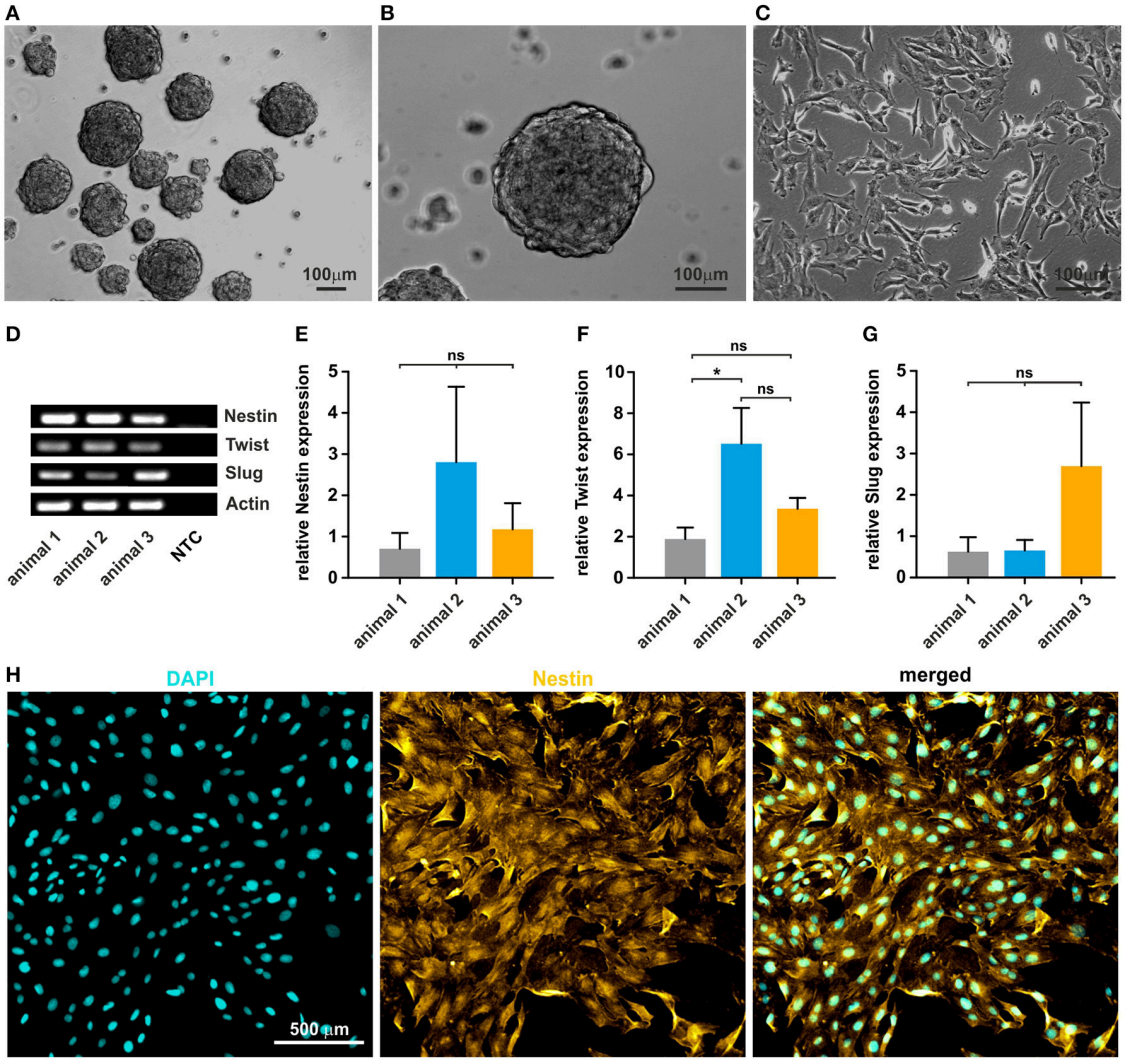
Isolated oNCSCs form neurospheres and show stem cell characteristics *in vitro*. **(A,B)** Cells isolated from the ovine sub-palatal tissue form neurospheres in serum-free culture conditions. Scale bar: 100 μm. **(C)** Dissociated oNCSCs can be expanded *in vitro* as adherent monolayer cultures. Scale bar: 100 μm. **(D)** oNCSCs express mRNA for the typical neural-crest markers Nestin, Twist, and Slug. NTC: no template control. Actin: housekeeping gene. **(E)** oNCSCs isolated from different animals express similar levels of Nestin, **(F)** Twist and **(G)** Slug. Data is shown as mean ± SEM from three independent experiments, ANOVA with Bonferroni correction, ^*^p < 0.5 was considered significant, CI 95%. **(H)** Immunocytochemistry revealed expression of Nestin (yellow) at protein level. A representative experiment is shown. Nuclear counterstaining was performed using DAPI (cyan). Scale bar: 50 μm.

After mechanical and enzymatic dissociation of the tissue, cultivated oNCSCs efficiently formed primary and secondary neurospheres (Figures 2A,B). After propagation as neurospheres, the clusters were dissociated and oNCSCs were further expanded as adherent culture (Figure 2C). Here, we were able to show that oNCSCs cultivated adherently exhibit a morphology similar to rodent and human NCSCs (Widera et al., 2009; Hauser et al., 2012). Next, we examined the stem cell character of oNCSCs by assessing the expression of typical neural crest cell markers *Slug, Twist*, and *Nestin*. RT-PCR revealed that oNCSCs isolated from three animals express *Slug, Twist*, and *Nestin* (Figure 2D). In order to assess potential differences in the expression level of the neural crest markers, additional quantitative RT-PCRs were performed.

No significant differences in the expression levels of *Nestin* and *Slug* were found and only slightly, but significantly higher expression of *Twist* mRNA in animal 2 (Figures 2E–G). In order to validate the expression of *Nestin* at protein level, oNCSCs were cultivated as adherent monolayer followed by immunocytochemical stainings. We were able to demonstrate that *Nestin* was expressed by most oNCSCs (Figure 2H).

### oNCSCs are highly proliferative and migratory

**Figure 3:**
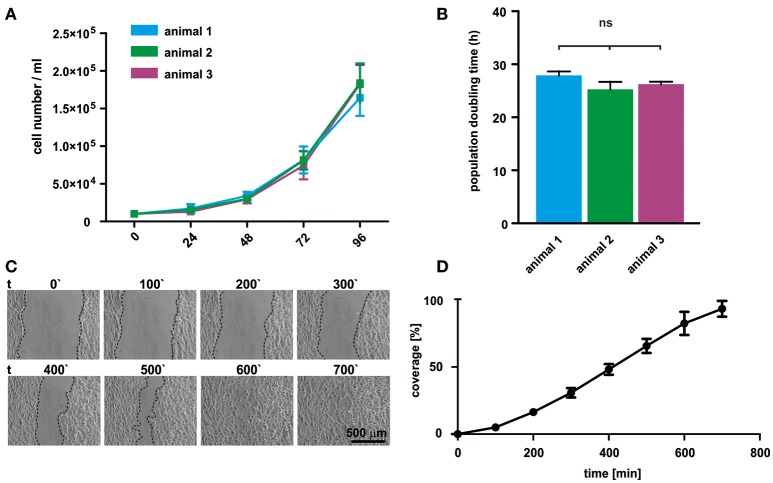
oNCSCs are highly proliferative and migrate *in vitro*. **(A)** Total cell number was determined after 24, 48, 72, and 96 h in adherent cultures of oNCSCs isolated from three different animals revealing normal exponential growth. **(B)** No significant differences in the population doubling time was observed between different oNCSC isolations. **(C)** *In vitro* wound healing assay was performed to assess the migratory potential of oNCSCs. Representative images are shown. Scale bar: 500 μm. **(D)** Scratch coverage was assessed over time. Full coverage was achieved between 9 and 11 h. All data are presented as mean ± SEM from three independent experiments.

Proliferation of oNCSCs from three individual animals was assessed by total cell number determination. The cell numbers were determined every 24 h revealing typical exponential growth without signs of spontaneous differentiation (Figure 3A). Moreover, no significant differences in the population doubling time were observed with an average doubling time ranging from 25 to 29 h (Figure 3B).

In order to assess the migratory behavior of oNCSCs, a wound healing assay was used. Briefly, a vertical scratch was set in confluent adherent oNCSC cultures and the migration was monitored over 12 h using time lapse microscopy (Figure 3C). Full closure of the scratch was observed between 9 and 11 h of migration (Figures 3C,D).

### oNCSCs can give rise to ectodermal and mesodermal progeny

**Figure 4:**
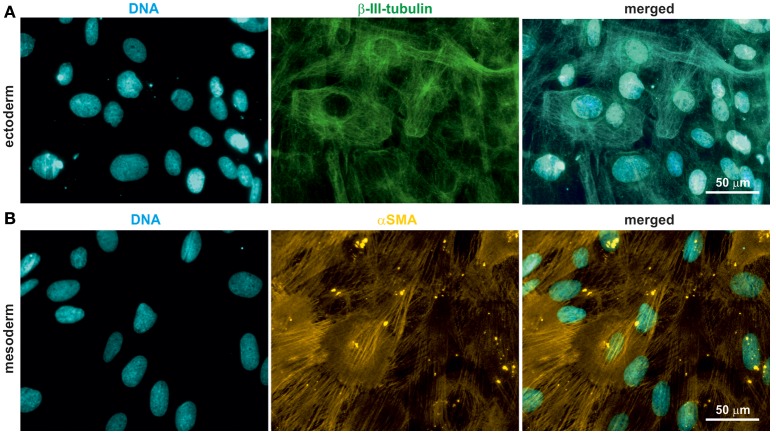
oNCSCs differentiate into cells of the ectodermal and mesodermal lineage. **(A)** oNCSCs were differentiated toward the ectodermal fate followed by immunocytochemical analysis. Cells subjected to ectodermal differentiation showed β-III-tubulin expression (middle panel). Nuclei were counterstained using DAPI. Scale bar: 50 μm. **(B)** Smooth muscle actin (αSMA) was expressed in oNCSCs after mesodermal differentiation. Nuclei were counterstained using DAPI. Scale bar: 50 μm.

Rodent and human NCSCs differentiate into mesodermal and ectodermal neural crest derivatives *in vitro* (Widera et al., 2009; Hauser et al., 2012; Müller et al., 2015). To test the ability of oNCSCs to undergo ectodermal and mesodermal differentiation, appropriate cultivation protocols were applied, followed by immunocytochemical stainings. We were able to show that oNCSCs differentiated toward the ectoderman fate homogenously expressed β-III-tubulin (Figure 4A). In addition, α smooth muscle actin (αSMA) expression was detected in oNCSCs differentiated toward the mesodermal fate (Figure 4B).

### oNCSCs differentiate into mesenchymal neural crest derivatives including osteogenic, adipogenic, and chondrogenic cells

**Figure 5:**
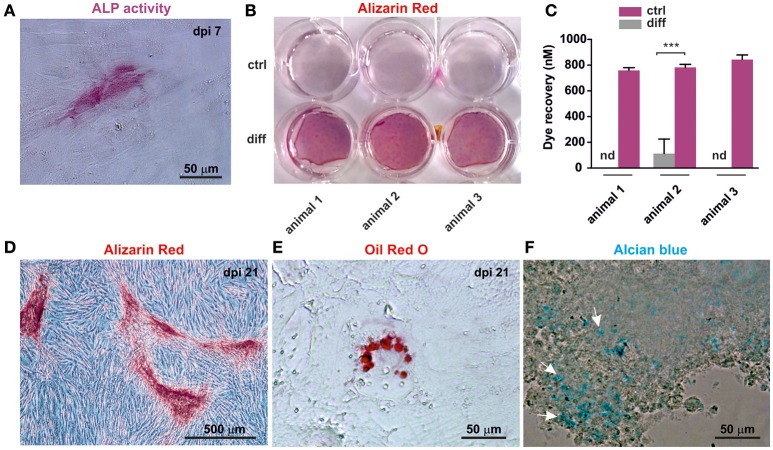
oNCSCs are capable of differentiating into osteogenic, adipogenic, and chondrogenic cell types. **(A)** oNCSCs cultivated in osteoinductive medium for 7 days show alkaline phosphatase (ALP) activity. Scale bar: 50 μm. **(B,C)** Alizarin red stain and dye recovery assays revealed that oNCSCs cultivated for 21 days in osteogenic differentiation medium (**B**: lower wells; **C**: magenta bars) deposit significantly higher levels of calcium deposits compared to control cells ^***^P < 0.0001. **(D)** Magnified picture of a representative culture of oNCSCs– derived osteocytes (21 days) stained with alizarin red. Scale bar: 500 μm. **(E)** oNCSCs differentiate into adipocytes as indicated by the presence of lipid droplets stained with Oil Red O. Scale bar: 50 μm. **(F)** After 21 days of chondrogenic differentiation, oNCSCs pellets express glycoproteins as indicated by alcian blue staining (arrows). Scale bar: 50 μm.

Cranial neural crest cells have the intrinsic ability to differentiate into osteogenic, adipogenic and chondrogenic cells. In order to test their ability to form these cell types, cultivated oNCSCs were subjected to directed differentiation protocols. oNCSCs differentiated toward the osteogenic fate exhibited ALP activity 7 days post induction (Figure 5A). Moreover, 21 days after the induction of osteogenesis, calcium deposits were detected using alizarin red staining (Figures 5B,D) and a significantly higher dye recovery was observed in cells cultured in differentiation medium compared to control cells in normal cultivation medium (Figure 5C). In the following, the ability of oNCSCs to undergo adipogenic differentiation was assessed. Here, Oil Red O-stained lipid droplets were evident in oNCSCs cultivated under differentiation conditions (Figure 5E) but not in control cells (not shown).

Finally, glycoprotein presence was evidenced by Alcian blue staining in oNCSC-pellets subjected to chondrogenic differentiation for 21 days (Figure 5F).

## Discussion

The mammalian oral cavity is an endogenous niche for various post-migratory NCSC populations (Widera et al., 2007, 2009; Pelaez et al., 2013; Boddupally et al., 2016; Fournier et al., 2016).

In the present study, we were able to show for the first time that similar to rodent and human palate, the ovine palatal lamina propria contains NCSCs. In particular, we found Nestin-positive oNCSCs within the Meissner corpuscles (Figure 1) underneath the epithelial layer. Notably, this distribution is similar to the palatal localization of NCSCs in rats (Widera et al., 2009) and mice (Widera et al., 2012) where numerous Nestin-positive NCSCs within Meissner corpuscles can be observed in the lamina propria of hard palate. Interestingly, in humans the number of Meissner corpuscles and consequently NCSCs gradually decreases with age (Schimrigk and Ruttinger, 1980). Thus, future studies investigating if this tendency can be observed in sheep could be of high interest for the regenerative medicine.

Formation of neurospheres in culture is an intrinsic property of NCSCs from different rodent and human origins including the periodontal ligament (Widera et al., 2007), oral mucosa (Abe et al., 2016; Fournier et al., 2016), palatal tissue (Widera et al., 2009), and nasal turbinates (Hauser et al., 2012). We successfully isolated ovine palatal NCSCs from the hard palate and were able to cultivate them as free floating neurospheres.

In our hands, cultivation of oNCSCs in serum-free medium supplemented with B27, FGF-2 and EGF resulted in a low proliferation rate (data not shown). This phenomenon is known for human NCSCs that proliferate significantly slower in serum-free NCSC medium compared to EGF and FGF-2-containing medium supplemented with human blood plasma (Greiner et al., 2011; Hauser et al., 2012). Thus, we modified the cultivation medium by supplementing the FGF-2 and EGF-containing medium with 10% newborn calf serum which is known to support growth and proliferation of murine neural crest cells (Sviderskaya et al., 2009). This medium composition resulted in an average population doubling time of 26 h without significant differences between individual oNCSC preparations (Figures 3A,B). This concords with the population doubling time of 25 h reported for human inferior turbinate-derived NCSCs (ITSCs) when cultivated in presence of human blood plasma (Greiner et al., 2011). Neural crest-derived olfactory epithelium MSCs exhibit a very similar population doubling time (~23 h, Veron et al., 2018). In general agreement with our observations in oNCSCs, human NCSCs cultivated in serum-free medium without blood plasma supplementation doubled within 175 h (Greiner et al., 2011). Interestingly, rat palatal NCSCs have been reported to double their population size within 65 h even in serum-free medium (Widera et al., 2009) suggesting differences in growth factor dependence between rat, ovine and human NCSCs.

In addition to Nestin, adult NCSCs post-migratory human and rodent NCSCs have been described to express a wide spectrum of embryonic neural crest markers including *Slug* and *Twist in vivo* and *in vitro* (see Kaltschmidt et al., 2012 for a full list of markers). Using RT-PCR and qPCR we demonstrated that oNCSC express *Nestin, Twist*, and *Slug* at mRNA level (Figures 2D–G). In addition, using immunocytochemistry, we were able to confirm the expression of Nestin at protein level (Figure 2H). The expression of these markers is well described for mouse, rat and human NCSCs (reviewed in Kaltschmidt et al., 2012).

One of the important features of early neural crest cells is their ability to migrate (Kaucka et al., 2016). Similarly, adult NCSCs show high migratory behavior *in vitro* and *in vivo* (Widera et al., 2007; Gosau et al., 2013; Keeve et al., 2013; Nishikawa et al., 2015). In this study, we were able to show that oNCSCs are highly migratory as evidenced in a wound healing assay (Figures 3C,D). During development, embryonic cranial neural crest cells migrate of out their intermediate niche underneath the ectoderms and differentiate in the following into ectodermal and mesodermal derivatives including peripheral neurons, melanocytes, as well as into osteogenic, and chondrogenic cells. This unique high developmental potential is only surpassed by pluripotent stem cells. Notably, post-migratory NCSCs retain at least some degree of this plasticity in adults making these cells ideal candidates for the use within regenerative medicine. In particular, ectodermal and mesodermal differentiation has been reported for various adult NCSC populations of rodent and human origin. In line with this findings, cultivation of oNCSCs under neurogenic conditions resulted in an up-regulation of the early ectodermal marker β-III-tubulin (Figure 4A). However, the morphology of the differentiated cells appeared immature with low levels of neuritic arborization indicating that longer differentiation time and optimization of the protocols for oNCSCs might be required to obtain mature neurons.

In contrast, conditions developed for osteogenic differentiation of MSCs resulted in robust osteogenesis in oNCSCs evidenced by an early ALP-activity (7 days after induction, Figure 5A) and mineralization 21 days after osteogenic induction (Figures 5B–D). Moreover, we detected differentiation of oNCSCs into adipogenic cells and chondrogenic cells if cultivated under appropriate conditions (Figures 5E,F). Mesenchymal differentiation is an intrinsic property of embryonic neural crest cells and post-migratory NCSCs. Although the osteogenic differentiation of non-embryonic NCSCs has been discussed controversially, a robust osteogenic differentiation of post-migratory NCSCs has been eloquently confirmed in protein zero-Cre/floxed-EGFP double transgenic mice (Urano-Morisawa et al., 2017).

In conclusion, we showed that the ovine hard palate contains post-migratory, Nestin expressing NCSCs. We successfully isolated and characterized oNCSCs and demonstrated that these cells express neural crest markers, and are highly proliferative and migratory *in vitro*. Finally, we differentiated oNCSCs into ectodermal and mesenchymal NCSC-derivatives. Taken together, our study could pave the way for the development of pre-clinical large animal models allowing middle- and long term study of safety and efficacy of endogenous NCSC transplantations.

## Author contributions

Conceived and designed the experiments: W-DG and DW. Performed the experiments: M-TZ, DH, ND, TM, W-DG, and DW. Analyzed the data: M-TZ, SS, W-DG, and DW. Wrote the paper: M-TZ, KP, W-DG, and DW.

### Conflict of interest statement

The authors declare that the research was conducted in the absence of any commercial or financial relationships that could be construed as a potential conflict of interest.
